# RNA-seq transcriptome analysis of the immature seeds of two *Brassica napus* lines with extremely different thousand-seed weight to identify the candidate genes related to seed weight

**DOI:** 10.1371/journal.pone.0191297

**Published:** 2018-01-30

**Authors:** Xinxin Geng, Na Dong, Yuquan Wang, Gan Li, Lijun Wang, Xuejiao Guo, Jiabing Li, Zhaopu Wen, Wenhui Wei

**Affiliations:** 1 College of Life Science and Technology, Henan Institute of Science and Technology / Collaborative Innovation Center of Modern Biological Breeding, Henan Province, Xinxiang, China; 2 Oil Crops Research Institute of the Chinese Academy of Agricultural Sciences/Key Laboratory of Biology and Genetic Improvement of Oil Crops, Ministry of Agriculture, Wuhan, China; 3 Applied Biotechnology Center, Wuhan Institute of Bioengineering, Wuhan, China; Huazhong University of Science and Technology, CHINA

## Abstract

*Brassica napus* is an important oilseed crop worldwide. Although seed weight is the main determinant of seed yield, few studies have focused on the molecular mechanisms that regulate seed weight in *B*. *napus*. In this study, the immature seeds of G-42 and 7–9, two *B*. *napus* doubled haploid (DH) lines with extremely different thousand-seed weight (TSW), were selected for a transcriptome analysis to determine the regulatory mechanisms underlying seed weight at the whole gene expression level and to identify candidate genes related to seed weight. A total of 2,251 new genes and 2,205 differentially expressed genes (DEGs) were obtained via RNA-seq (RNA sequencing). Among these genes, 1,747 (77.61%) new genes and 2020 (91.61%) DEGs were successfully annotated. Of these DEGs, 1,118 were up-regulated and 1,087 were down-regulated in the large-seed line. The Kyoto Encyclopedia of Genes and Genomes (KEGG) database analysis indicated that 15 DEGs were involved in ubiquitin-mediated proteolysis and proteasome pathways, which might participate in regulating seed weight. The Gene Ontology (GO) database indicated that 222 DEGs were associated with the biological process or molecular function categories related to seed weight, such as cell division, cell size and cell cycle regulation, seed development, nutrient reservoir activity, and proteasome-mediated ubiquitin-dependent protein catabolic processes. Moreover, 50 DEGs encoding key enzymes or proteins were identified that likely participate in regulating seed weight. A DEG (*GSBRNA2T00037121001*) identified by the transcriptome analysis was also previously identified in a quantitative trait locus (QTL) region for seed weight via SLAF-seq (Specific Locus Amplified Fragment sequencing). Finally, the expression of 10 DEGs with putative roles in seed weight and the expression of the DEG *GSBRNA2T00037121001* were confirmed by a quantitative real-time reverse transcription PCR (qRT-PCR) analysis, and the results were consistent with the RNA sequencing data. This work has provided new insights on the molecular mechanisms underlying seed weight-related biosynthesis and has laid a solid foundation for further improvements to the seed yield of oil crops.

## Introduction

*Brassica napus* is an important oilseed crop that is used as an edible oil and as an animal feed worldwide. In recent years, this crop has been popularly used as an ornamental plant and renewable energy source in China [1]. In addition to silique number per plant and seed number per silique, seed weight is one of the three major components of yield for oilseed rape [2]. Increasing the seed weight is the primary approach to improve the yield of *B*. *napus* [2]. Only on chromosome A09, 17 QTL were identified for seed weight [3], which showed the genetic complexity of seed weight regulation. Therefore, understanding the regulatory mechanisms underlying gene expression and selecting the appropriate candidate genes for seed weight are of great significance for yield improvement in oilseed rape breeding.

However, few reports have focused on the molecular mechanisms that regulate the differences of seed weight in *B*. *napus*, which may be related to complicated quantitative characteristics or environment [4]. Recently, the genome sequence of *B*. *napus* has been reported, and it can be utilized to study the regulatory mechanisms underlying seed weight [2]. Several seed weight (size) genes have been successfully cloned, such as those in rice and *Arabidopsis thaliana*. In *B*. *napus*, Liu *et al*. [5] reported that variations in the *auxin-response factor 18* (*ARF18*) genes on chromosome A9 could regulate the seed weight without changing the pod, and it represents the first polyploidy crop yield gene acquired by map-based cloning. The molecular mechanism underlying yield genes has frequently been studied in rice, and the results provide rich information for our study on the seed weight of *B*. *napus*. Many genes related to grain weight and size have been reported in rice, including *GW2*, *GW5*, *GL3*, and *GS5*. *GW2* and *GW5* have similar functions in regulating grain weight. Song et al. [6] revealed that *GW2* encodes a ring-type E3 ubiquitin ligase. Loss of *GW2* function regulates cell division and increases the cell number, enlarges the spikelet hull, accelerates the grain milk-filling rate, and results in enhanced grain width, weight and yield. Weng *et al*. [7] found that *GW5* most likely regulates cell division during grain development through the ubiquitin-proteasome pathway. There are three key enzymes required for the ubiquitin-proteasome pathway: E1, E2 and E3 [8]. Ubiquitin is initially activated by E1, and then E2 and E3 work in conjunction to recognize the substrate protein and conjugate it to ubiquitin. Ubiquitin is then easily attached as a previously synthesized chain or a monomer. Finally, the ubiquitinated protein is transported to the proteasome for degradation [9]. Qi *et al*. [10] noted that *GL3*.*1* encodes Ser/Thr phosphatase, a member of the protein phosphatase kelch (PPKL) family, and found that *GL3*.*1* accelerates cell division by influencing protein phosphorylation in the spikelet, thereby resulting in longer grains and higher yields in rice. Further studies showed that *GL3*.*1* directly dephosphorylates its substrate Cyclin-T1;3. The down-regulation of Cyclin-T1;3 in rice results in a shorter grain, which occurs through a novel phosphatase-mediated process during cell cycle progression. *GS5* encodes a putative serine carboxypeptidase that acts as a positive regulator of cell division. The high expression of *GS5* promotes and accelerates the cell cycle, and it then accelerates cell division of the outer glume and ultimately enlarges the grain size and increases the grain weight [11]. In *A*. *thaliana*, the cloned genes primarily regulated cell division, although they also controlled the cell number or cell size of the integument, embryo, and endosperm and affected the final seed weight or seed size. Du *et al*. [12] reported that *SUPPRESSOR2 OF DA1* (*SOD2*) encodes ubiquitin-specific protease15 (UBP15), which regulates seed size by promoting cell division in the integuments of ovules and developing seeds. *DA1* and *DA2* encode a ubiquitin receptor and a RING-type protein with E3 ubiquitin ligase activity, respectively, which determine the final seed weight and organ size by restricting the period of cell proliferation [13,14]. The *ARF2* gene links auxin signalling, cell division, and the final size of seeds and other organs [15]. Jofuku *et al*. [16] revealed that *APETALA2* (*AP2*) plays an important role in determining the seed size, seed weight and seed oil and protein accumulation, and it acts via the maternal sporophyte and endosperm genomes to control seed weight and seed yield. Previous research on seed weight has provided helpful information for our work identifying candidate genes for seed weight via transcriptome analyses.

Next-generation sequencing (NGS) technology has been widely used in biological research. Because the genomes of a large number of valuable species have been published, NGS technology has rapidly developed [17]. The transcriptome is the set of all messenger RNA and non-coding RNA molecules in one cell or a population of cells for a specific development stage or physiological condition. Transcriptome analysis is an important method of studying and exploring functional genes in plants. Compared with genome analyses, transcriptome analyses only assess transcribed genes; thus, they have a smaller research scope, which may produce more pertinent results [18]. In recent years, RNA-seq technology has rapidly developed into the most important method for transcriptome profiling [19]. Plant transcriptome analyses could provide considerable information on highly expressed genes, differentially expressed genes and new genes [20–22]. Transcriptome sequencing has been widely applied in studies on *Brassica*, such as transcriptome profile analysis of young floral buds of fertile and sterile plants [23], transcriptome profiling of resistance to pathogen [24] and research to narrow down the number of candidate genes in identified quantitative trait locus (QTL) regions [25].

In this study, two transcriptomes were compared for the immature seeds of two *B*. *napus* lines with extremely different thousand-seed weight (TSW). A total of 2,251 new genes and 2,205 (differentially expressed genes) DEGs were obtained. A bioinformatics analysis was performed to investigate those differentially expressed genes related to seed weight. A DEG (*GSBRNA2T00037121001*) involved in the identified QTL region for seed weight is an important candidate gene for further seed weight research. These databases will provide an important resource and helpful insights for identifying candidate genes related to seed weight and improving the final yield of *B*. *napus*.

## Results and discussion

### Comparison of the seed weight, seed size and fresh seed weight at different seed stages between two extreme lines

**Fig 1:**
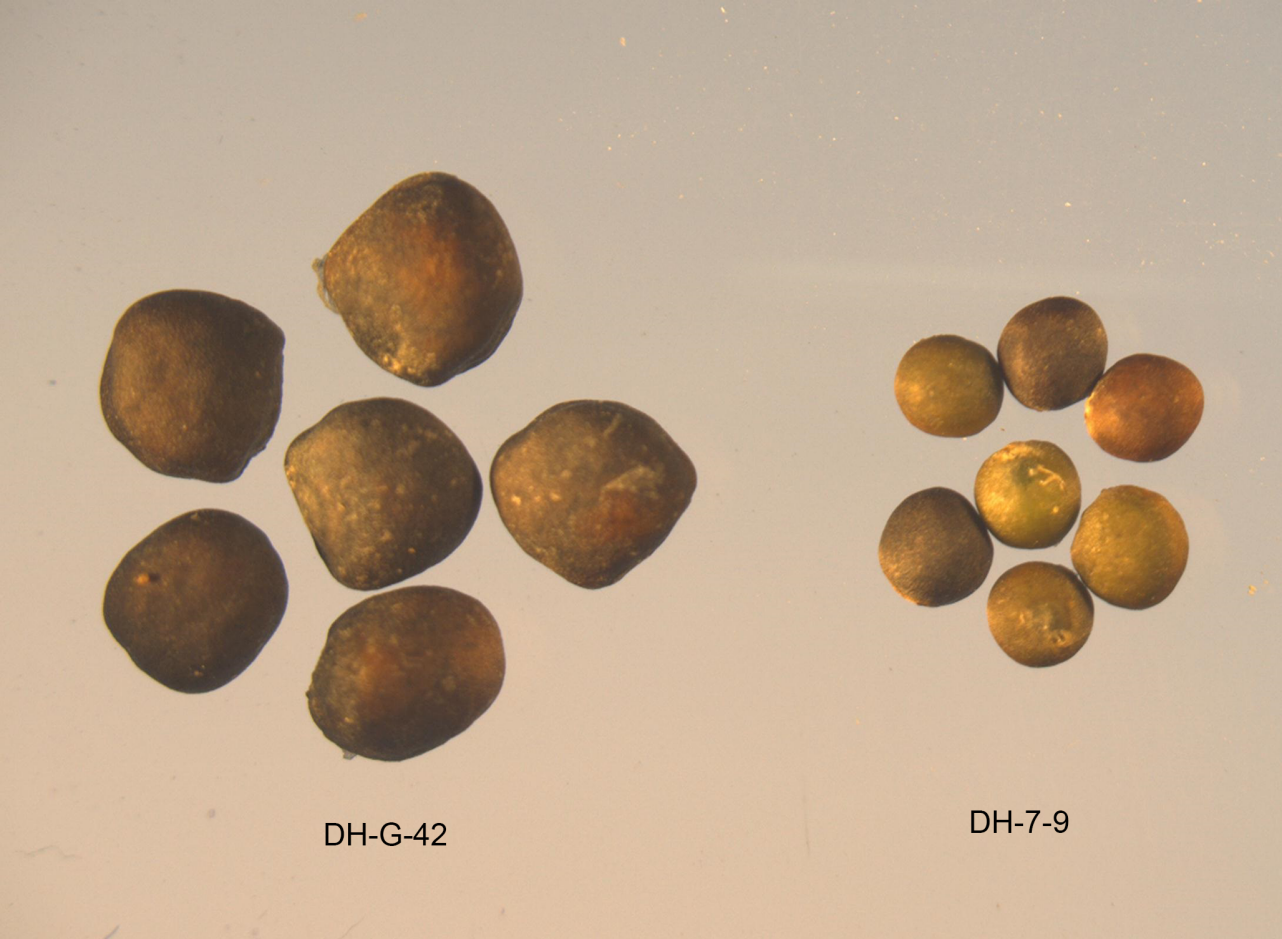
The seed phenotype of two extreme *B*. *napus* lines (large-seed line DH-G-42 and small-seed line DH-7-9).

The results showed that the seed size and seed weight of the large-seed line DH-G-42 were significantly greater than those of the small-seed line DH-7-9 (Fig 1, Table 1). The statistical data for the fresh seed weight revealed that there were few differences between the large-seed line and the small-seed line during 1 and 2 weeks after flowering (WAF). However, from 3 to 6 weeks after flowering, the fresh weight of DH-G-42 was obviously higher than that of DH-7-9 (Table 1). Thus, the 3^rd^ week might be the key period in which the two lines differentiate. In *B*. *napus*, from 20 days after flowering to maturity was the fastest growing period for seed development, which supported our results.

**Table 1:** Statistics of the seed weight, seed size and fresh seed weight at different periods of development.

Sample ID	Thousand seed weight (g)	Seed length (mm)	Fresh seed weight (mg)					
			1 WAF	2 WAF	3 WAF	4 WAF	5 WAF	6 WAF
**DH-G-42**	6.2±0.02 a*	2.5±0.10 a*	1.58±0.01 e	1.46±0.04 e	3.68±0.10 d	7.03±1.04 c	12.98±1.39 b	14.62±0.42 a*
**DH-7-9**	2.45±0.08 b	1.6±0.26 b	1.07±0.07 e	1.15±0.11 e	1.17±0.06 e	2.64±0.06 de	3.8±0.26 d	4.22±0.17 d

* Means followed by the same lowercase letters are not significantly different at P ≤ 0.05 by Tukey’s Honest Significant Differences test.

### Transcriptome sequencing

After the construction and sequencing of two cDNA libraries, a total of 9.06 G and 7.16 G nucleotides were generated from T3 (large-seed) and T4 (small-seed) libraries (named by the Sequencing Co.), respectively (Table 2). After removing the adaptor sequences, low quality and short reads, 7.16 G and 6.88 G data were obtained for large seed line and small seed line, respectively. The N content was 0% in both libraries. The Q30 percentage exceeded 90%, and the GC (guanine-cytosine) content was 46.57% and 47.38% for the large-seed and small-seed lines, respectively, which suggests that the sequencing data were highly accurate and reliable. The total reads of large (70,844,224) and small seed line (67,971,356) were blasted with the reference genome of *B*. *napus*. On average, 82.04% and 82.75% of the reads were successfully mapped to the reference genome by Tophat (v2.0.12) (Table 3).

**Table 2:** Comparison of the raw transcriptome data for the large-seed line and small-seed line in *B*. *Napus*.

Sample	Total nucleotides (G)	Used nucleotides (G)	GC (%)	N (%)	^a^ Q20 (%)	Cycle Q20 (%)	^b^ Q30 (%)
**T3**	9.06	7.16	46.57	0	98.79	100	93.91
**T4**	8.68	6.88	47.38	0	98.79	100	93.87

^a^ Q20 indicates a quality score of 20, a 1% chance of error and 99% confidence

^b^ Q30 indicates a quality score of 30, a 0.1% chance of error and 99.9% confidence

**Table 3:** Statistics of RNA-seq data aligned to the reference genome.

Sample	Total reads	Total mapping reads	Uniquely mapping reads	Multiple mapping reads	Pair-mapped reads	Single mapped reads
**T3**	70,844,224	58,121,707 (82.04%)	52,338,904 (90.05%)	5,782,803 (9.95%)	40,002,696 (68.83%)	6,512,313 (11.2%)
**T4**	67,971,356	56,247,370 (82.75%)	50,709,768 (90.15%)	5,537,602 (9.85%)	38,609,476 (68.64%)	5,933,668 (10.55%)

### Analysis of new genes and differentially expressed genes (DEGs)

After filtering the genes that only contained one exon or encoded short peptide chains (less than 50 amino acid residues), a total of 2,251 new genes and 97,963 genes were revealed by blasting the reference genome using Cufflinks. The sequences and detailed information are listed in S1 and S2 Tables.

**Fig 2:**
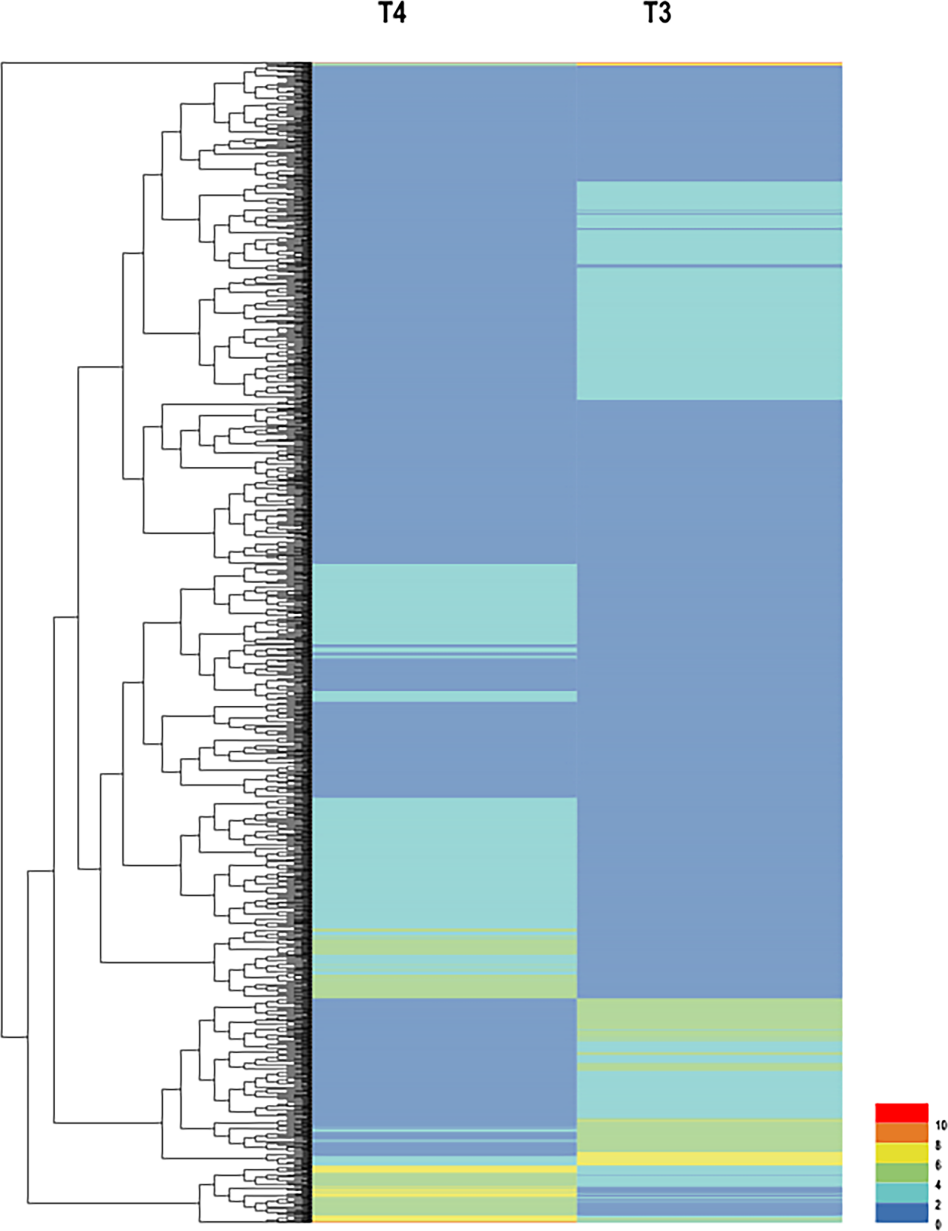
Hierarchical clustering analysis of DEGs based on FPKM data between T3 and T4. The colour key represents the FPKM (Fragments Per Kilobase of exon per Million fragments mapped) normalized log_2_ transformed counts. Red represents high expression, and blue represents low expression. Each row represents a gene.

To identify the DEGs between the large-seed and small-seed samples, the Fragments Per Kilobase of exon per Million fragments mapped (FPKM) was used to calculate the gene expression levels. The results showed that a total of 2,205 genes were differentially expressed between two libraries, of which, 1,118 genes were up-regulated and 1,087 genes were down-regulated in T3 compared with T4. Detailed information is listed in S3 Table. The up-regulated and down-regulated genes between T3 and T4 were revealed by a hierarchical clustering analysis. The results of the hierarchical clustering analysis based on the FPKM values (Fig 2). It was found that about 29% DEGs with the log^2^FPKM value was lower than 2, 63% DEGs with the log^2^FPKM value was between 2 and 6, 8% DEGs had the log^2^FPKM value between 6 and 10, and one DEG (*GSBRNA2T00002721001*) owned the log^2^FPKM value up to 11.89. In Fig 2, all gene expression levels seemed to be low, that is because we set the reference level of DEGs’ log2FPKM value (10) was much higher than normal (6) between T3 and T4. Furthermore, there were too many genes expressed during the seed development, a higher reference level facilitated to screen the differentially expressed genes between T3 and T4.

### Functional annotations

**Fig 3:**
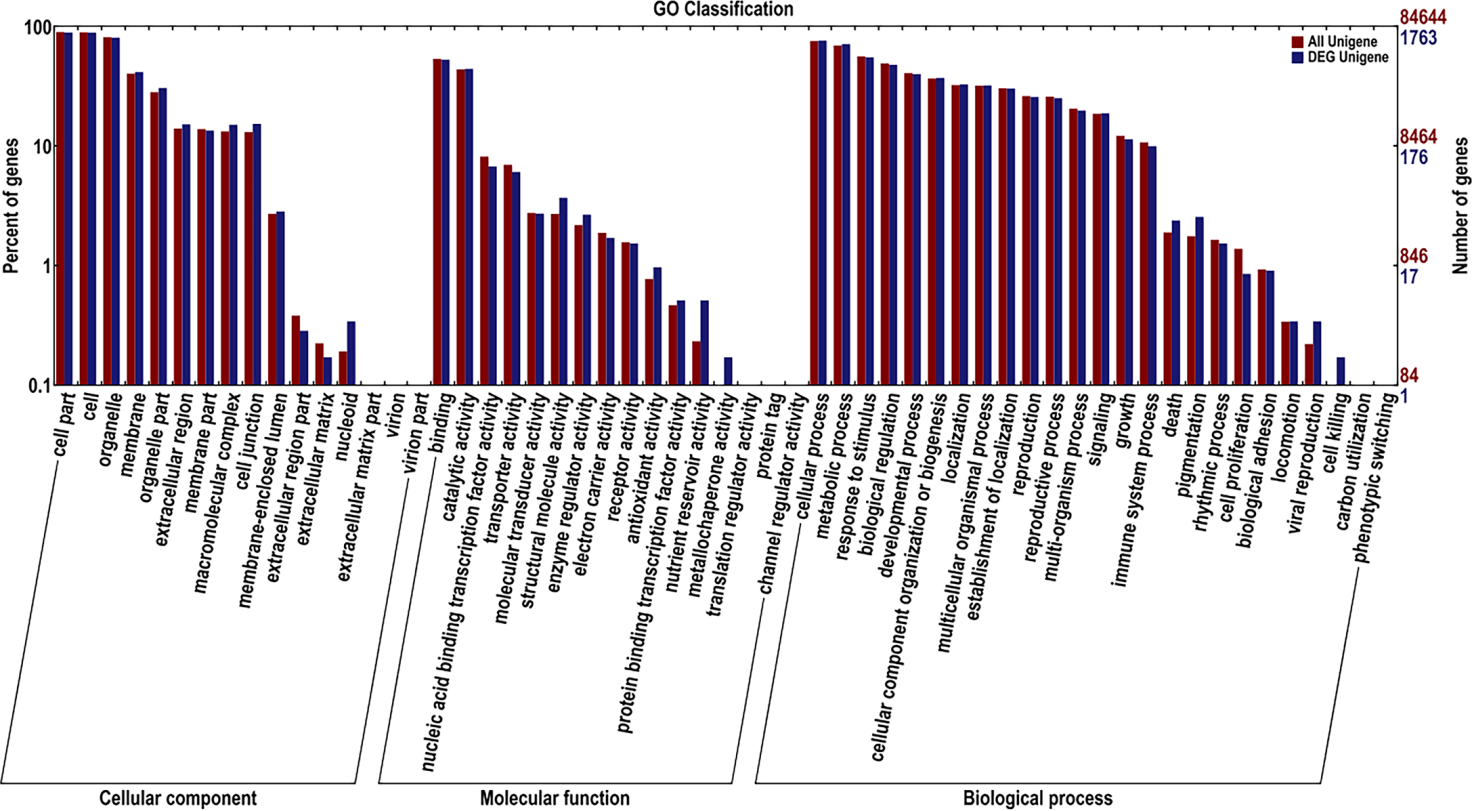
Gene ontology (GO) function classification of differentially expressed genes (DEGs) in *B*. *napu*s. A total of 1,763 genes were assigned to the three main GO functional categories and then divided into 57 sub-categories.

To functionally annotate the *B*. *napus* seed transcriptome, the 2,251 new genes and 2,205 DEGs were applied in a blast search of the GO (Gene Ontology), COG (Cluster of Orthologous Group), KEGG (Kyoto Encyclopedia of Genes and Genomes), Swiss-Prot and NR (non-redundant) databases using BLAST (Basic Local Alignment Search Tool) software, which successfully annotated 1,747 new genes and 2,020 DEGs. All of the annotated information is listed in Table 4 and S4 Table. Of these new genes, 1,733, 1,069, 1,321, 336 and 276 were annotated in the GO, COG, KEGG, Swiss-Prot and Nr databases, respectively. For the GO classification analysis of DEGs, 1,763 genes were assigned to three main GO functional categories and then divided into 57 sub-categories (Fig 3). Several DEGs were assigned to more than one sub-category. We then calculated the percentage of DEGs involved in each sub-category (S5 Table). The sub-category with largest percentage in the “cellular component” category was cell part (DEGs accounted for 89.17% of all genes involved in this category), followed by cell (88.94%) and organelle (80.77%). The sub-category with the largest percentage in the “molecular function” category was binding (52.98%) and then catalytic activity (44.36%). The sub-category with the largest percentage in the “biological process” category was cellular process (76.52%), followed by metabolic process (71.47%), response to stimulus (55.42%) and biological regulation (47.87%). These results indicated that the primary cause resulting in different seed weight might be related to the differential expression of genes in these nine sub-categories, which would provide a direction for further analysis.

**Table 4:** Functional annotation of new genes and differentially expressed genes in the COG, GO, KEGG, Swiss-Prot and NR databases.

Annotation Database	Number of Annotated New Genes	300< = Length<1000	Length> = 1000	Number of Annotated DEGs
**COG_Annotation**	276 (12.26%)	146	121	678 (30.75%)
**GO_Annotation**	1,321 (58.69%)	681	614	1,763 (79.95%)
**KEGG_Annotation**	336 (14.93%)	191	135	493 (22.38%)
**Swissprot_Annotation**	1,069 (47.49%)	520	526	1,451 (65.8%)
**NR_Annotation**	1,733 (76.99%)	882	813	2,015 (91.38%)
**All_Annotated**	1,747 (77.61%)	894	815	2,020 (91.61)

**Fig 4:**
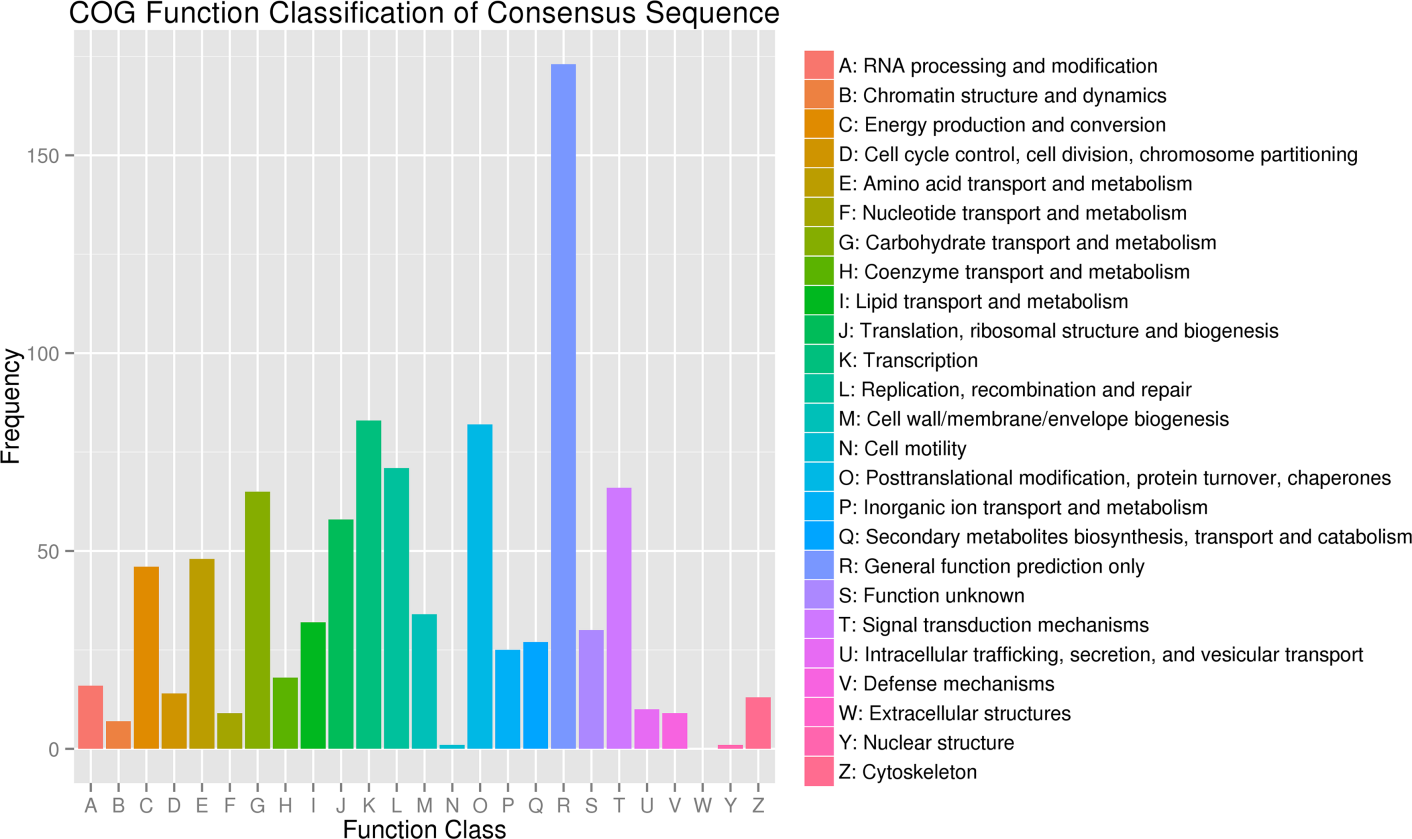
Clusters of orthologous groups (COG) function classification of differentially expressed genes (DEGs) in *B*. *napu*s. A total of 678 DEGs were distributed across 25 COG categories.

The COG functional classification analysis showed that the DEGs were distributed across 25 COG categories (Fig 4). We also calculated the percentage of DEGs in each category (S6 Table) and found that the largest percentage was “General function prediction only” (20.69%), followed by “Transcription” (12.24%), “Posttranslational modification, protein turnover, chaperones” (12.09%) and “Replication, recombination and repair” (10.47%). Many genes were differentially expressed in these categories, which might explain the different seed weights in the large-seed and small-seed lines.

Information on the metabolic pathways of the transcriptome is valuable for understanding the physiological processes of seed development after flowering. To evaluate the differences between the two lines, the metabolic pathways of the DEGs were analyzed by classification. An analysis using the KEGG database on biological pathways showed that a total of 493 genes were assigned to 97 pathways (Table 4; S7 Table). However, the number of genes involved in these 97 pathways was 505 instead of 493, which suggests that certain genes might be involved in more than one KEGG pathway, such as the gene “*Brassica_newGene_5407*”, which is involved in cysteine and methionine metabolism; glycine, serine and threonine metabolism; and lysine biosynthesis. Among the 97 pathways, the largest pathway was ribosome, which contained 31 genes, followed by spliceosome (19), protein processing in endoplasmic reticulum (18), oxidative phosphorylation (15) and plant hormone signal transduction (14).

Approximately 91% (2,015) and 66% of the DEGs (1,451) were successfully annotated using the NCBI's NR database and the Swiss-Prot database, respectively (Table 4).

### Identification of seed weight-related genes in the *B*. *napus* transcriptome and QTL region

**Fig 5:**
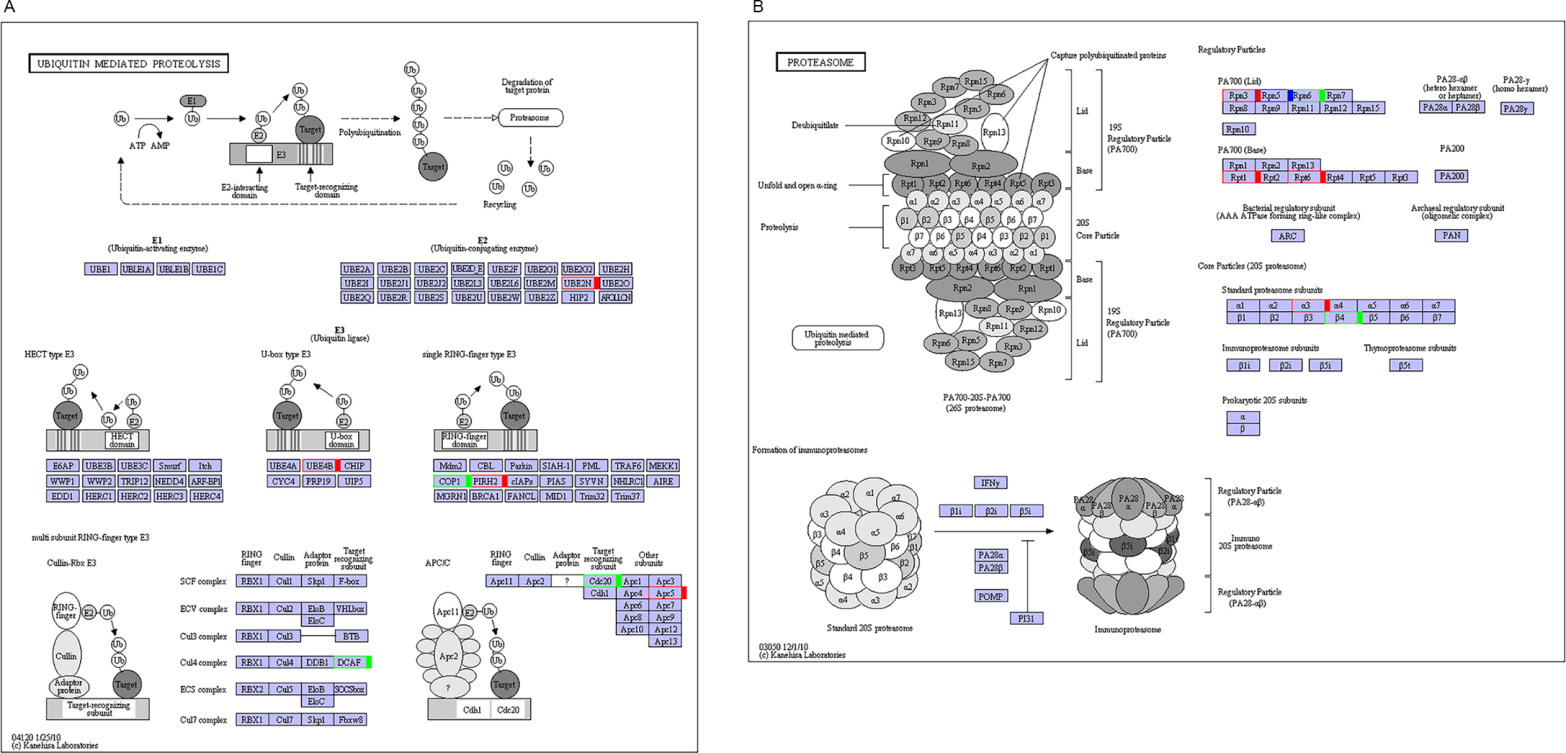
KEGG pathway analysis identified the ubiquitin-mediated proteolysis pathway (ko04120) and proteasome pathway (ko03050) for 15 DEGs between T3 and T4. (A) ko04120, and (B) ko03050.

**Fig 6:**
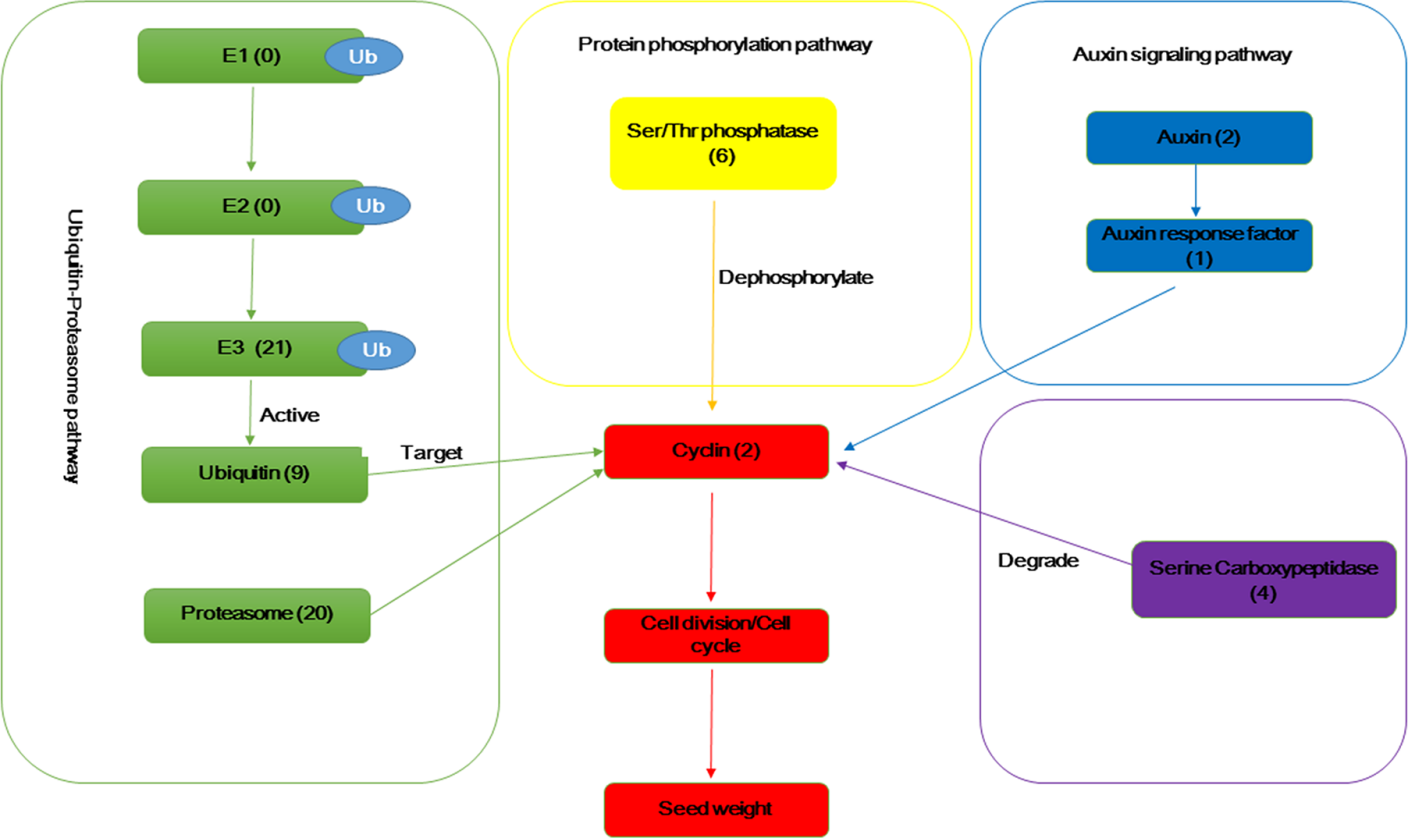
The putative pathways of the differentially expressed genes (DEGs) involved in seed weight formation. The values in the bracket indicate the number of DEGs. Ub, Ubiquitin; E1, Ubiquitin activating enzyme; E2, Ubiquitin conjugating enzyme; E3, Ubiquitin ligase.

Information on the seed weight (seed size) genes in rice, *A*. *thaliana* and *B*. *napus* were used to study the pathways, biological processes, key enzymes and proteins related to cell division, cell cycle, ubiquitin-proteasome, auxin response factor, Ser/Thr phosphatase, cyclin, and serine carboxypeptidase. The KEGG pathway analysis identified 6 and 9 DEGs for the ubiquitin-mediated proteolysis (ko04120) (Fig 5A) and proteasome (ko03050) (Fig 5B) pathways, respectively (Table 5). Analysis of the related enzymes and proteins of seed weight in the Swiss-Prot and NR databases (Table 6) revealed that 35 DEGs (24 up-regulated and 11 down-regulated) were annotated as encoding the key enzymes and proteins of ubiquitin-protease biological processes. Two genes encoded cell cycle-related enzymes and protein cyclin-dependent kinases and cyclin, and one up-regulated gene encoded the auxin response factor. Four genes (1 up-regulated and 3 down-regulated) encoded serine carboxypeptidase, and 6 genes (4 up-regulated and 2 down-regulated) encoded serine/threonine-protein phosphatase and its regulatory subunit or isozyme. The putative pathways of the above DEGs involved in seed weight formation were shown in Fig 6.

**Table 5:** Summary of DEGs related to seed weight (ubiquitin-mediated proteolysis pathway and proteasome pathway) in the KEGG database between T3 and T4.

Pathway type	Pathway ID	DEG number	Up/Down (numbers of genes)	Gene ID
**Ubiquitin-mediated proteolysis**	ko04120	6	4 Up	GSBRNA2T00022870001
				GSBRNA2T00031738001
				GSBRNA2T00114729001
				GSBRNA2T00151756001
			2 Down	GSBRNA2T00018127001
				GSBRNA2T00025567001
**Proteasome**	ko03050	9	5 Up	Brassica_newGene_1807
				GSBRNA2T00063942001
				GSBRNA2T00074567001
				GSBRNA2T00116823001
				GSBRNA2T00134599001
			4 Down	GSBRNA2T00150716001
				GSBRNA2T00048424001
				Brassica_newGene_2120
				GSBRNA2T00108467001

**Table 6:** Summary of key enzymes and proteins related to seed weight in the Swiss-Prot and NR databases.

Enzymes/protein	DEG number	Up/Down (numbers of genes)	Gene ID
**Cyclin-dependent kinase inhibitor 3**	1	1 Down	*GSBRNA2T00109531001*
**Cyclin-B1-2**	1	1 Down	*GSBRNA2T00051807001*
**E3 ubiquitin ligase**	2	2 Up	*GSBRNA2T00140145001; GSBRNA2T00097655001*
**E3 ubiquitin-protein ligase**	19	14 Up, 5 Down	*GSBRNA2T00049900001; GSBRNA2T00073497001;* *GSBRNA2T00080006001; GSBRNA2T00076273001;* *GSBRNA2T00052859001; GSBRNA2T00062381001;* *GSBRNA2T00125575001; GSBRNA2T00136480001;* *GSBRNA2T00017196001; GSBRNA2T00018260001;* *Brassica_newGene_5236; GSBRNA2T00063271001;* *GSBRNA2T00018008001; GSBRNA2T00032349001;* *GSBRNA2T00011257001; GSBRNA2T001195860011;* *Brassica_newGene_1243; GSBRNA2T00151834001;* *GSBRNA2T00097032001*
**Ubiquitin-protein ligase 4**	2	2 Up	*GSBRNA2T00119586001; GSBRNA2T00017196001*
**Ubiquitin family protein**	1	1 Down	*GSBRNA2T00123522001*
**26S proteasome non-ATPase regulatory subunit**	5	2 Up, 3 Down	*GSBRNA2T00150716001; GSBRNA2T00108467001;* *Brassica_newGene_1807; Brassica_newGene_2120;* *GSBRNA2T00134599001*
**26S protease regulatory subunit**	6	4 Up, 2 Down	*GSBRNA2T00109634001; GSBRNA2T00074567001;* *GSBRNA2T00116823001; GSBRNA2T00018348001; GSBRNA2T00151849001; Brassica_newGene_5455*
**Auxin response factor 4**	1	1 Up	*Brassica_newGene_719*
**Serine carboxypeptidase**	3	3 Down	*GSBRNA2T00105277001; GSBRNA2T00158449001;* *GSBRNA2T00136613001*
**Serine carboxypeptidase S28 family protein**	1	1 Up	*GSBRNA2T00001408001*
**Serine/threonine-protein phosphatase PP1 isozyme**	2	1 Up, 1 Down	*Brassica_newGene_1210; GSBRNA2T00046355001*
**Serine/threonine protein phosphatase regulatory subunit**	3	2 Up, 1 Down	*GSBRNA2T00026535001; GSBRNA2T00109497001;* *GSBRNA2T00080035001*
**Serine/threonine-protein phosphatase 7 GN**	1	1 Up	*Brassica_newGene_4770*
**Auxin-responsive protein IAA12**	1	1 Up	*GSBRNA2T00025700001*
**IAA-amino acid hydrolase 2**	1	1 Down	*GSBRNA2T00060750001*

On chromosome A09 of *B*. *napus*, a hot QTL region of ~0.58 Mb between nucleotides 25,401,885 and 25,985,931 was identified to contain 91 candidate genes associated with seed weight according to SLAF-seq in our previous studies [2]. The information for these 91 candidate genes is presented in S8 Table. A comparison of the 91 candidate genes obtained by SLAF-seq and the 2,205 DEGs in the *B*. *napus* seed transcriptome identified one gene (*GSBRNA2T00037121001*) that is involved in inorganic ion transport and metabolism and encodes a ferritin according to the annotated information. Identification of the DEGs in QTL regions can substantially narrow down the number of candidate genes. The annotation of all these genes in further analyses will provide important information.

### Quantitative real-time reverse transcription PCR (qRT-PCR) analysis of DEGs

**Fig 7:**
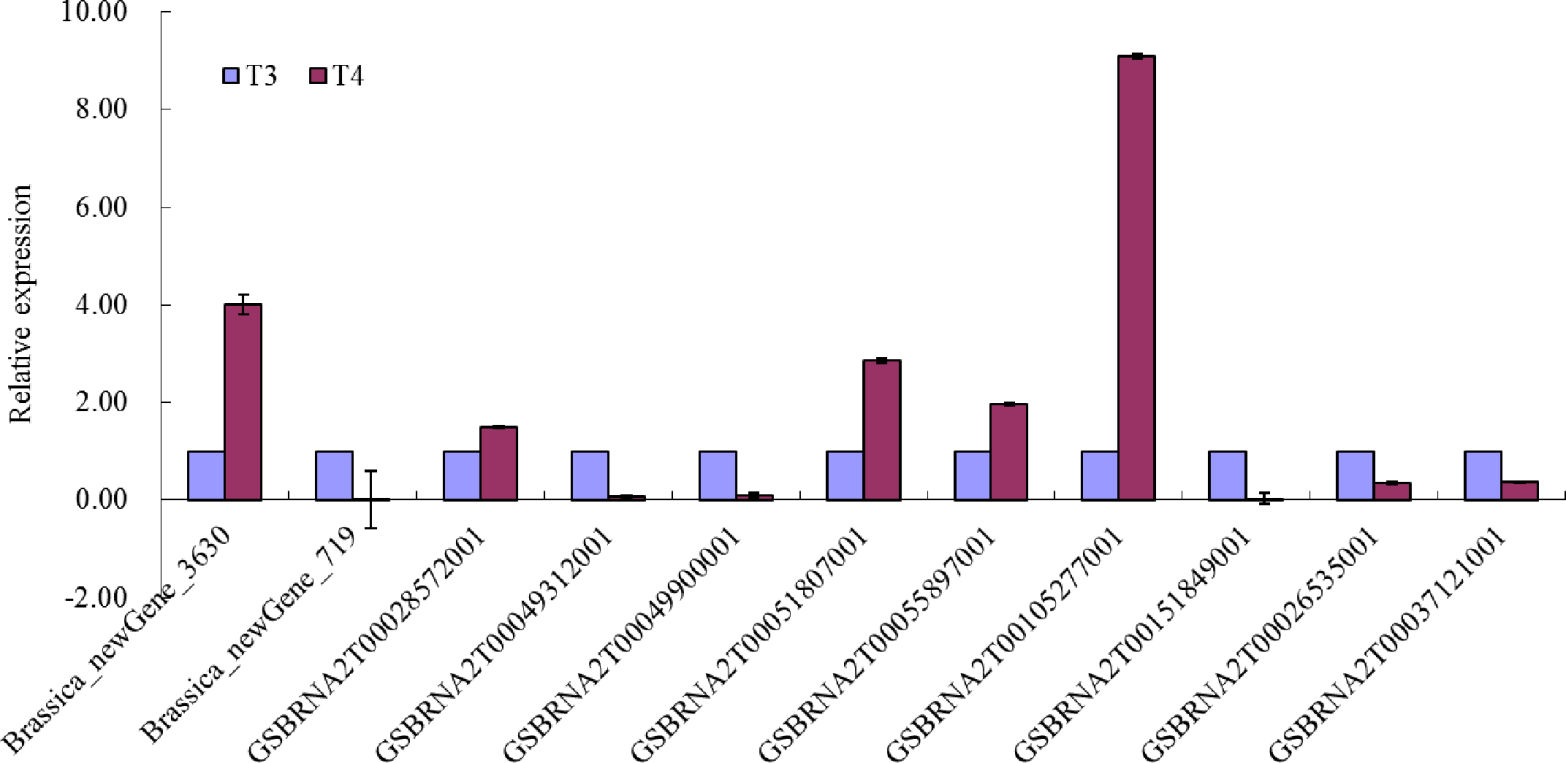
Relative expression of 11 differentially expressed genes (DEGs) related to seed weight between two extreme *B*. *napus* lines via qRT-PCR. Ten DEGs encoding key enzymes or proteins related to seed weight and one DEG in the QTL region were analyzed via qRT-PCR to validate the transcriptome.

To verify the expression level of DEGs detected by RNA-seq, 10 DEGs that were predicted to encode the key enzymes or proteins related to seed weight and the DEG *GSBRNA2T00037121001* in the QTL region of TSW were selected for qRT-PCR validation. Among these 10 randomly selected DEGs, 5 genes were up-regulated (higher expression levels in T3 than T4), whereas the remaining 5 genes were down-regulated (lower expression levels in T3 than T4) (Fig 7). The DEG in the QTL region was still up-regulated as shown by the transcriptome analysis. The data obtained by the qRT-PCR was consistent with the RNA-seq results, which suggests the reliability of the transcriptome database.

## Conclusions

In this study, RNA-seq technology was used to reveal the immature seed transcriptome of two extremely different lines (large-seed line and small-seed line) in *B*. *napus*. A total of 2,251 new genes and 2,205 DEGs were identified. According to the analysis of annotated information, the identified candidate genes related to seed weight mainly encoded the key enzymes or proteins. A DEG (*GSBRNA2T00037121001*) that was previously identified in the QTL region for seed weight might be an important candidate gene for further seed weight research in *B*. *napus*. Several important DEGs were analyzed by qRT-PCR to verify the RNA-seq results. This work has provided new insights into the molecular mechanisms underlying seed weight-related biosynthesis and has laid a solid foundation for further improvements to the seed yield of oil crops. Further annotation of those new genes and DEGs will also provide rich information and a reference transcriptome for future genome-wide gene expression research in *B*. *napus*.

## Materials and methods

### Plant materials

In 2005, a large-seeded and a small-seeded *B*. *napus* germplasms were collected. And a few progeny lines from cross hybridization between these two germplasms were identified with heavier thousand-seed weight than large-seed parent, and some progeny lines had lighter TSW than small-seed parent. Then two progeny lines with extremely different TSW were selected to perform microspore culture to obtain their DH lines, named DH-G-42 and DH-7-9. DH-G-42 and DH-7-9 were planted and grown under non-stressed conditions from October 2014 to May 2015 in a field at the experimental farm of the Oil Crops Research Institute of the Chinese Academy of Agricultural Sciences, Wuhan, China. The flowering time of DH-G-42 and DH-7-9 began on 12 March 2015 and 2 March 2015, respectively. Immature seeds at each time point from 1 to 6 weeks after flowering were obtained from the lines DH-G-42 and DH-7-9, frozen in liquid nitrogen immediately, and stored at—80°C.

### Phenotypic observations

The seeds of three plants from the two lines were respectively harvested to measure the seed weight and seed size at the yielding time. The samples were oven dried at 80°C to constant weight. In this experiment, the thousand seed weight of each line was evaluated as the means of three replicated weights of 1000 seeds. The seed size was measured from the mean values of the diameter of three seeds of each line, with three replications. The fresh weight of immature seeds at each time point from 1 to 6 weeks after flowering were determined by the mean values of 100 seeds, with three replicates of each line.

### RNA extraction, cDNA library construction and sequencing

Total RNA was isolated from the seeds at six different stages from 1 to 6 weeks after flowering using TRIzol reagent (Invitrogen, Carlsbad, CA, USA) according to the manufacturer’s instructions. RNase-free DNase I was used to remove the residual DNA for 30 min at 37°C. The extracted RNA was qualified and quantified using a NanoDrop 2000 UV–Vis spectrophotometer (NanoDrop, Wilmington, DE, USA) and the samples showed a 260/280 nm ratio between 1.8 and 2.2 and an OD260/230 > 1.0. For each line, equal RNA samples at each stage, including three replicates, were pooled for the RNA-seq.

The mRNA-seq libraries were prepared using the Illumina TruSeq RNA Sample Preparation Kit (Illumina Inc., San Diego, CA, USA), and the mRNA isolation, fragment interruption and RNA-seq were performed by the Biomarker Technologies Corporation according to their standard protocol. Ultimately, the libraries were constructed for sequencing using the Illumina HiSeqTM 2500 sequencing platform (Illumina Inc., San Diego, CA, USA) at Biomarker Technologies Corporation in Beijing. The adaptor sequences, empty reads and low quality sequences were filtered from the raw reads. Real-time monitoring was performed for each cycle during sequencing and the ratio of high-quality reads with quality scores greater than Q30 (indicating a quality score of 30 and a 0.1% chance of an error; 99.9% confidence) for the raw reads and guanine-cytosine (GC) content was calculated for quality control.

### Identification of new genes and differentially expressed genes (DEGs)

Tophat (v2.0.12) is a fast mapping tool for RNA-seq reads that can identify splice junctions between exons [26]. Cufflinks can be used to assemble the reads into transcripts based on the mapping results [27]. The software programs Tophat and Cufflinks were used to blast the sequencing reads with the reference genome of *B*. *napus* (1.2 Gb, download link: http://www.genoscope.cns.fr/brassicanapus/data/ [28]) for the differential gene and transcript expression analyses. This process could reveal new genes that have not been previously annotated using reference genomes and could also evaluate the abundance of gene expression. The Fragments Per Kilobase of exon per Million fragments mapped (FPKM) method was used to calculate the abundance of gene expression. DESeq [29] is suitable for analysing biological duplicate samples obtained from DEG screening, and EBSeq [30] is suitable for non-biological duplicate samples. During the DEG screening, a false discovery rate (FDR) <0.001 and fold change> = 8 were considered standard values. Here, the FDR was obtained via a P-value correction and considered the key index of the differential gene expression analysis.

Fold-change formula:

$$Fold\;change=log_{2}\;(normalized\;expression\;level\;of\;DEG\;in\;T4/T3)$$

First, the fold change and FDR were calculated from the normalized expression. If the DEG fold change is > = 8, then a FDR <0.001 indicates that the DEG was significantly different between T3 and T4.

### Functional annotation

Gene ontology (GO) is the de facto standard for gene functionality descriptions, and it is widely used in functional annotations and enrichment analyses [31]. The Cluster of Orthologous Groups of proteins (COG) database is based on the phylogenetic relationships among bacteria, algae and eukaryotes. Gene products in an orthologous relationship can be classified using the COG database [32]. In an organism, different genes coordinate to exert biological functions. Pathway analyses are helpful for further interpreting gene functions. The Kyoto Encyclopedia of Genes and Genomes (KEGG) database is the main public database on pathways [33]. In this study, a functional enrichment analysis of new genes and differentially expressed genes was conducted by performing a blast search against the GO, COG, KEGG pathway, Swiss-Prot [34] and Non-redundant protein (NR) [35] databases using BLAST software [36].

### Quantitative real-time reverse transcription PCR (qRT-PCR) analysis

The transcript levels of ten candidate DEGs regulating seed weight were also verified by qRT-PCR. Total RNA [1 μg, pre-treated with DNase I (Promega, Madison, USA)] was reverse transcribed using a reverse transcriptase (Promega, Madison, USA). A 5 μl aliquot of 1:20 diluted cDNA was used as the template in a 20 μl PCR system. The qRT-PCR was performed using SYBR green reaction mix (SYBR Green qRT-PCR Master Mix; Toyobo) after a pre-incubation at 95°C for 5 min, followed by 40 cycles of denaturation at 95°C for 15 s, annealing at 60°C for 15 s, and extension at 72°C for 32 s in an ABI PRISM7500 Sequence Detection System (Applied Biosystems). The 18S rRNA was used as the internal standard because it is uniformly expressed in *B*. *napus* tissue. All reactions were performed using one biological sample with three technical replicates. The comparative Ct method was used for the data analysis. Primer pairs were designed using Primer 5 software, and the primer sequences are available in S9 Table.

### Statistical analysis

The data were analyzed using a statistical package, SPSS version 16.0 (SPSS, Chicago, IL, USA). The variation between DH-G-42 and DH-7-9 in thousand mature seed weight, mature seed length, and fresh seed weight during seed development (1 to 6 weeks) was evaluated by analysis of variance (ANOVA) followed by Tukey’s Honest Significant Differences test. All statistical analysis data had three repeats. Results were considered significant at P ≤ 0.05.

## Supporting information

S1 Table The list of all genes by RNA-seq. (XLSX)

S2 Table The list of new genes by RNA-seq. (XLSX)

S3 Table The list of differentially expressed genes (DEGs) by RNA-seq. (XLSX)

S4 Table The annotated information of DEGs. (XLSX)

S5 Table The GO classification of DEGs. (XLSX)

S6 Table The COG classification of DEGs. (XLSX)

S7 Table KEGG pathway annotation of DEGs. (XLSX)

S8 Table Annotated information for 91 candidate genes. (XLSX)

S9 Table Primers for quantitative real-time PCR. (XLSX)

## References

[1] Xie L, Li P, Zhang W, Yang M. A review on the development potential of bioenergy rapeseed. Chinese J Bioprocess Eng. 2005; 3: 28–31.

[2] Geng X, Jiang C, Yang J, Wang L, Wu X, Wei W. Rapid identification of candidate genes for seed weight using the SLAF-Seq method in *Brassica napus*. PLoS One. 2016; 11: e0147580 doi: 10.1371/journal.pone.0147580 26824525 10.1371/journal.pone.0147580 PMC4732658

[3] Fu Y, Wei D, Dong H, He Y, Cui Y, Mei J, et al Comparative quantitative trait loci for silique length and seed weight in *Brassica napus*. Sci Rep. 2015; 5: 14407 doi: 10.1038/srep14407 26394547 10.1038/srep14407 PMC4585775

[4] Zhao W, Wang X, Wang H, Tian J, Li B, Chen L, et al Genome-wide identification of QTL for seed yield and yield-related traits and construction of a high-density consensus map for QTL comparison in *Brassica napus*. Front Plant Sci. 2016; 7: 17 doi: 10.3389/fpls.2016.00017 26858737 10.3389/fpls.2016.00017 PMC4729939

[5] Liu J, Hua W, Hu Z, Yang H, Zhang L, Li R, et al Natural variation in ARF18 gene simultaneously affects seed weight and silique length in polyploid rapeseed. Proc Natl Acad Sci USA. 2015; 112: E5123–E5132. doi: 10.1073/pnas.1502160112 26324896 10.1073/pnas.1502160112 PMC4577148

[6] Song XJ, Huang W, Shi M, Zhu MZ, Lin HX. A QTL for rice grain width and weight encodes a previously unknown RING-type E3 ubiquitin ligase. Nat Genet. 2007; 39: 623–630. doi: 10.1038/ng2014 17417637 10.1038/ng2014

[7] Weng J, Gu S, Wan X, Gao H, Guo T, Su N, et al Isolation and initial characterization of GW5, a major QTL associated with rice grain width and weight. Cell Res. 2008; 18: 1199–1209. doi: 10.1038/cr.2008.307 19015668 10.1038/cr.2008.307

[8] Depre C, Powell SR, Wang X. The role of the ubiquitin-proteasome pathway in cardiovascular disease. Cardiovasc Res. 2010; 85: 251–252. doi: 10.1093/cvr/cvp362 19892772 10.1093/cvr/cvp362

[9] Stangl K, Stangl V. The ubiquitin-proteasome pathway and endothelial (dys) function. Cardiovasc Res. 2010; 85: 281–290. doi: 10.1093/cvr/cvp315 19767293 10.1093/cvr/cvp315

[10] Qi P, Lin YS, Song XJ, Shen JB, Huang W, Shan JX, et al The novel quantitative trait locus *GL3*.*1* controls rice grain size and yield by regulating cyclin-T1;3. Cell Res. 2012; 22: 1666–1680. doi: 10.1038/cr.2012.151 23147796 10.1038/cr.2012.151 PMC3515756

[11] Li Y, Fan C, Xing Y, Jiang Y, Luo L, Sun L, et al Natural variation in *GS5* plays an important role in regulating grain size and yield in rice. Nat Genet. 2011; 43: 1266–1269. doi: 10.1038/ng.977 22019783 10.1038/ng.977

[12] Du L, Li N, Chen L, Xu Y, Li Y, Zhang Y, et al The ubiquitin receptor DA1 regulates seed and organ size by modulating the stability of the ubiquitin-specific protease UBP15/SOD2 in *Arabidopsis*. Plant Cell. 2014; 26: 665–677. doi: 10.1105/tpc.114.122663 24585836 10.1105/tpc.114.122663 PMC3967032

[13] Li Y, Zheng L, Corke F, Smith C, Bevan MW. Control of final seed and organ size by the DA1 gene family in *Arabidopsis thaliana*. Genes Dev. 2008; 22:1331–1336. doi: 10.1101/gad.463608 18483219 10.1101/gad.463608 PMC2377187

[14] Xia T, Li N, Dumenil J, Li J, Kamenski A, et al The ubiquitin receptor DA1 interacts with the E3 ubiquitin ligase DA2 to regulate seed and organ size in *Arabidopsis*. Plant Cell. 2013; 25: 3347–3359. doi: 10.1105/tpc.113.115063 24045020 10.1105/tpc.113.115063 PMC3809536

[15] Schruff MC, Spielman M, Tiwari S, Adams S, Fenby N, Scott RJ. The *AUXIN RESPONSE FACTOR 2* gene of *Arabidopsis* links auxin signalling, cell division, and the size of seeds and other organs. Development. 2006; 133: 251–261. doi: 10.1242/dev.02194 16339187 10.1242/dev.02194

[16] Jofuku KD, Omidyar PK, Gee Z, Okamuro JK. Control of seed mass and seed yield by the floral homeotic gene *APETALA2*. Proc Natl Acad Sci USA. 2005; 102: 3117–3122. doi: 10.1073/pnas.0409893102 15708974 10.1073/pnas.0409893102 PMC549499

[17] Mardis ER. Next-generation DNA sequencing methods. Annu Rev Genomics Hum Genet. 2008; 9: 387–402. doi: 10.1146/annurev.genom.9.081307.164359 18576944 10.1146/annurev.genom.9.081307.164359

[18] Szabo DT. Transcriptomic biomarkers in safety and risk assessment of chemicals. Biomarkers in Toxicology. 2014; 1033–1038.

[19] Wang Z, Gerstein M, Snyder M. RNA-Seq: a revolutionary tool for transcriptomics. Nat Rev Genet. 2009; 10: 57–63. doi: 10.1038/nrg2484 19015660 10.1038/nrg2484 PMC2949280

[20] Loraine AE, McCormick S, Estrada A, Patel K, Qin P. RNA-Seq of *Arabidopsis* pollen uncovers novel transcription and alternative splicing. Plant Physiol. 2013; 162: 1092–1109. doi: 10.1104/pp.112.211441 23590974 10.1104/pp.112.211441 PMC3668042

[21] Gao Y, Xu H, Shen Y, Wang J. Transcriptomic analysis of rice (*Oryza sativa*) endosperm using the RNA-Seq technique. Plant Mol Biol. 2013; 81: 363–378. doi: 10.1007/s11103-013-0009-4 23322175 10.1007/s11103-013-0009-4

[22] Jones SI, Vodkin LO. Using RNA-Seq to profile soybean seed development from fertilization to maturity. PLoS One. 2013; 8: e59270 doi: 10.1371/journal.pone.0059270 23555009 10.1371/journal.pone.0059270 PMC3598657

[23] Yan XH, Dong CH, Yu JY, Liu WH, Jiang CH, Liu J, et al Transcriptome profile analysis of young floral buds of fertile and sterile plants from the self-pollinated offspring of the hybrid between novel restorer line NR1 and *Nsa* CMS line in *Brassica napus*. BMC Genomics. 2013; 14: 26 doi: 10.1186/1471-2164-14-26 23324545 10.1186/1471-2164-14-26 PMC3556089

[24] Xing M, Lv H, Ma J, Xu D, Li H, Yang L, et al Transcriptome profiling of resistance to *Fusarium oxysporum* f. sp. conglutinans in cabbage (*Brassica oleracea*) roots. PLoS One. 2016; 11: e0148048 doi: 10.1371/journal.pone.0148048 26849436 10.1371/journal.pone.0148048 PMC4744058

[25] Xu HM, Kong XD, Chen F, Huang JX, Lou XY, Zhao JY. Transcriptome analysis of *Brassica napus* pod using RNA-Seq and identification of lipid-related candidate genes. BMC Genomics. 2015; 16: 858 doi: 10.1186/s12864-015-2062-7 26499887 10.1186/s12864-015-2062-7 PMC4619414

[26] Trapnell C, Pachter L, Salzberg SL. TopHat: discovering splice junctions with RNA-Seq. Bioinformatics. 2009; 25: 1105–1111. doi: 10.1093/bioinformatics/btp120 19289445 10.1093/bioinformatics/btp120 PMC2672628

[27] Trapnell C, Williams BA, Pertea G, Mortazavi A, Kwan G, van Baren MJ, et al Transcript assembly and quantification by RNA-Seq reveals unannotated transcripts and isoform switching during cell differentiation. Nat Biotechnol. 2010; 28: 511–515. doi: 10.1038/nbt.1621 20436464 10.1038/nbt.1621 PMC3146043

[28] Chalhoub B, Denoeud F, Liu S, Parkin IA, Tang H, Wang X, et al Plant genetics. Early allopolyploid evolution in the post-Neolithic *Brassica napus* oilseed genome. Science. 2014; 345: 950–953. doi: 10.1126/science.1253435 25146293 10.1126/science.1253435

[29] Wang L, Feng Z, Wang X, Wang X, Zhang X. DEGseq: an R package for identifying differentially expressed genes from RNA-seq data. Bioinformatics. 2010; 26: 136–148. doi: 10.1093/bioinformatics/btp612 19855105 10.1093/bioinformatics/btp612

[30] Leng N, Dawson JA, Thomson JA, Ruotti V, Rissman AI, Smits BM, et al EBSeq: an empirical Bayes hierarchical model for inference in RNA-seq experiments. Bioinformatics. 2013; 29: 1035–1043. doi: 10.1093/bioinformatics/btt087 23428641 10.1093/bioinformatics/btt087 PMC3624807

[31] Ashburner M, Ball CA, Blake JA, Botstein D, Butler H, Cherry JM, et al Gene Ontology: tool for the unification of biology. Nat Genet. 2000; 25: 25–29. 10.1038/75556 PMC3037419 10802651

[32] Tatusov RL, Galperin MY, Natale DA, Koonin EV. The COG database: a tool for genome-scale analysis of protein functions and evolution. Nucleic Acids Res. 2000; 28: 33–36. 10592175 10.1093/nar/28.1.33 PMC102395

[33] Kanehisa M, Goto S, Kawashima S, Okuno Y, Hattori M. The KEGG resource for deciphering the genome. Nucleic Acids Res. 2004; 32: D277–D280. doi: 10.1093/nar/gkh063 14681412 10.1093/nar/gkh063 PMC308797

[34] Apweiler R, Bairoch A, Wu CH, Barker WC, Boeckmann B, Ferro S, et al UniProt: the universal protein knowledgebase. Nucleic Acids Res. 2004; 32: D115–D119. doi: 10.1093/nar/gkh131 14681372 10.1093/nar/gkh131 PMC308865

[35] Deng YY, Li JQ, Wu SF, Zhu YP, Chen YW, He FC. Integrated nr database in protein annotation system and its localization. Comput Eng. 2006; 32: 71–74.

[36] Altschul SF, Madden TL, Schäffer AA, Zhang J, Zhang Z, Miller W, et al Gapped BLAST and PSI-BLAST: a new generation of protein database search programs. Nucleic Acids Res. 1997; 25: 3389–3402. 9254694 10.1093/nar/25.17.3389 PMC146917

