# Correction: RNA-seq transcriptome analysis of the immature seeds of two *Brassica napus* lines with extremely different thousand-seed weight to identify the candidate genes related to seed weight

**DOI:** 10.1371/journal.pone.0218914

**Published:** 2019-06-25

**Authors:** Xinxin Geng, Na Dong, Yuquan Wang, Gan Li, Lijun Wang, Xuejiao Guo, Jiabing Li, Zhaopu Wen, Wenhui Wei

The Data Availability statement is incorrect. The underlying data are not provided in the published paper and its Supporting Information files. The authors have deposited the data in the Sequence Read Archive (accession numbers: SRR9165867, SRR9165099).
